# Parathyroid Paranoia: Unveiling Psychosis in Hyperparathyroidism

**DOI:** 10.1155/2024/8126125

**Published:** 2024-06-24

**Authors:** Rachael J. Murphy, Subin Paul, Ralph Primelo

**Affiliations:** ^1^ Department of Psychiatry Lehigh Valley Health Network, Bethlehem, Pennsylvania, USA; ^2^ Morsani College of Medicine University of South Florida, Tampa, Florida, USA

## Abstract

Primary hyperparathyroidism (PHPT) and subsequent hypercalcemia have been reported to be associated with psychosis. Here we report the case of a 28-year-old cannabis using male with his first contact with psychiatric care because of mood instability, bizarre behavior, and poor ability to carry out activities of daily living. Hypercalcemia was identified, and a subsequent endocrine workup confirmed PHPT. After parathyroidectomy, there was no longer any need for antipsychotic or other psychotropic medications; the report emphasizes the importance of considering organic causes, such as hyperparathyroidism, in patients presenting with psychotic-like symptoms, including in the setting of substance use disorder. Prompt recognition and appropriate management of the underlying condition are crucial for optimizing patient outcomes.

## 1. Introduction

Psychosis is a complex psychiatric condition that can arise from various etiologies, including primary psychiatric disorders, substance abuse, environmental factors, and organic causes [1]. Hyperparathyroidism, characterized by excessive production of parathyroid hormone and resultant hypercalcemia, is a rare but important organic cause of psychosis. The manifestation of symptoms often varies by the degree of hypercalcemia. Primary hyperparathyroidism (PHPT) has traditionally been recognized by its characteristic symptoms, including urolithiasis (“stones”); osteopenia and osteoporosis (“bones”); abdominal cramping, peptic ulceration (“moans”); and depression, anxiety, cognitive dysfunction, insomnia, confusion, and behavioral changes (“psychiatric overtones”). Mild cases may cause cognitive changes, anxiety, or depression, whereas more severe hypercalcemia may engender altered mental status or psychosis [2]. In patients undergoing parathyroidectomy for primary hyperparathyroidism, a common etiology of hypercalcemia, the prevalence of hallucinations and delusions ranged from 5.0% to 20.0%, and the prevalence of impaired cognition ranged from 37.3% to 46.5% [3]. These data support an association between hypercalcemia and neuropsychiatric changes. Here we present the case of a young man searching psychiatry for the first time with psychosis, which was likely caused by PHPT, and where the psychotic symptoms disappeared, and no antipsychotics were necessary after parathyroidectomy.

## 2. Case Presentation

The patient was a 28-year-old male with no prior psychiatric treatment who was brought to the hospital at the end of November 2022 by his wife due to mood instability, psychosis with bizarre behavior, and impaired activities of daily living for three weeks. The patient had a history of a similar episode 10 years prior while in college, which resolved after a period of sleep without any need for psychiatric treatment. The patient, who held a degree in physics, was employed in an entry-level engineering position at the time of presentation though had not been working for 1 month. Psychosocial stressors, including the recent loss of his mother, his wife's pregnancy, relocation from the northwestern to the northeastern United States in 2020, and lack of social supports, were observed. Family history included alcohol abuse and bipolar disorder in his mother. The patient denied suicidal or homicidal ideation, auditory or visual hallucinations, and reported feeling safe in the hospital.

Collateral information was gathered from the patient's cousin which provided insights into the patient's recent behavioral changes. Since early November 2022, the patient displayed sporadic and agitated behavior; the patient had been observed yelling and cursing at his family, which was uncharacteristic of him. He endorsed racing thoughts involving what the patient deemed “philosophical symbols,” while expressing himself with tangential speech and bizarre, repetitious phrases.

The patient's cousin had not seen the patient for 2 years, and he reported a significant decline in functioning compared to what the patient used to achieve. The patient's wife corroborated these observations. In addition, she mentioned that 10 years prior, when studying at college, the patient had similar behavior that lasted for approximately 1 week and resolved following a period of restful sleep. The recent episode appeared to severely impact the patient's emotional well-being, as he appeared visibly distressed and socially withdrawn during a holiday gathering with the family, in stark contrast to his typically outgoing nature.

During his voluntary hospitalization, the patient exhibited disorganized thinking and behavior, which was characterized by a heightened sense of suspicion and paranoia toward both staff and peers on the unit. He often stated angrily that the hospital was attempting to poison him. He closely observed other patients and nurses throughout the day. Initially, the patient refrained from participating in group therapy due to a general uneasiness in social interactions. For 1 week, he required redirection as he would stand very closely behind staff in the halls, while occasionally following other patients into their rooms, demonstrating a lack of boundaries. When questioned about these behaviors, the patient exhibited limited insight into their inappropriateness. He expressed experiencing unusual and intense ideas about “an important symbol,” and an overall absence of emotional responses. While he frequently mentioned his adherence to mathematical symbols since admission, he paradoxically struggled to articulate his current emotional state. The patient was also noted to display rapid blinking during conversations with staff at times. The patient was diagnosed with Bipolar 1 disorder, current episode manic, with psychotic behavior, although we wanted to determine whether an underlying medical condition may be contributing to these symptoms.

While on the inpatient psychiatric unit, the patient was initiated on olanzapine on December 1, which was titrated up to 20 mg nightly by December 7 to manage his mood and psychotic symptoms. Additionally, lorazepam was prescribed at 0.5 mg twice daily on December 7 to alleviate anxiety. Due to the severity and unusual nature of his initial presentation, his hospitalization ultimately resulted in his classification as an involuntary commitment. However, the patient's overall condition improved, as he soon expressed that he was feeling “pretty good.” His sleep patterns and appetite returned to normal by December 13, and he confidently denied experiencing depression, anxiety, or psychotic symptoms. Demonstrating active engagement, the patient then participated in various group activities. He maintained regular communication with his wife and father, although he did continue to struggle with a sense of poverty of content and accurately evaluating his own emotional state. Despite these challenges, he consistently adhered to his prescribed medication regimen and remained open to adjustments as deemed necessary by the treatment team. He was discharged by the inpatient unit on December 15, 2022.

Attention was also given to the patient's substance use history, with the recognition that his urine drug screen was positive for cannabinoids. It was documented the patient had been using cannabis for over 10 years. In the context of his overall treatment, it was discussed that the ongoing use of cannabis may contribute to the reoccurrence or exacerbation of psychiatric symptoms. He was strongly advised to abstain from drug use, with a recommendation for ongoing follow-up with an outpatient substance use disorder treatment provider.

**Figure 1:**
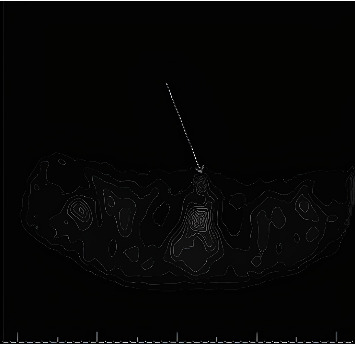
SPECT scan of parathyroid showing a focus of activity at the left thoracic inlet.

During his medical workup, it was discovered that the patient exhibited chronic hypercalcemia, with plasma calcium levels measuring at 12.5 (reference range, 8.5–10.5) mg/dL corresponding to 3.12 (reference range, 2.12–2.62) mmol/L. Notably, the patient had a history of kidney stones documented since 2021, which may have been related to underlying endocrine dysfunction. Further laboratory investigations revealed elevated levels of parathyroid hormone (PTH) at 112.7 (reference range, 10–65) pg/mL, and a toxicology screen positive for cannabinoids. His thyroid stimulating hormone (TSH), lipid profile, kidney function, electrolytes, white blood cell counts, and platelets, were otherwise within normal limits. Given these findings, an endocrinology consultation was sought, resulting in a recommendation for a parathyroid scan to confirm the suspected diagnosis of PHPT. Following consultation with the endocrinology team on December 9, cinacalcet was initiated at 30 mg each evening with dinner, and a single-photon emission computed tomography (SPECT) scan of his parathyroid was performed which showed a focus of activity at the left thoracic inlet, confirming PHPT (Figure 1). To aid in the diagnostic evaluation and treatment planning process, a computed tomography (CT) scan of the patient's head and a magnetic resonance imaging (MRI) scan of his brain were conducted. Inferiorly within the fourth ventricle, there were two small areas of calcification both measuring approximately 7 mm. The imaging results turned out to be unchanged from a prior MRI investigation performed in 2021 following a neurology consultation for chronic finger paresthesia and tongue numbness. At that time, the patient was recommended to see an endocrinologist, but he did not show up. Following his inpatient psychiatric stay, outpatient surgery consultation was advised to explore potential treatment options for hypercalcemia. He ultimately followed up in the endocrinology clinic and was operated with parathyroidectomy in August 2023.

Postsurgery the serum calcium levels normalized. It is reasonable to assume that with normalized calcium levels, PTH levels would also normalize, although this is not explicitly mentioned in the patient's chart. Furthermore, the patient was mentally significantly improved.

Since there is no mention of the patient continuing with olanzapine or any other psychiatric medication postsurgery, it can be inferred that he did not require ongoing medication for psychiatric symptoms.

The patient did not follow-up with outpatient psychiatry, and there were no new refills of psychiatric medications for over 6 months after his inpatient stay. Additionally, there was no information provided regarding the patient's use of cannabinoids postsurgery, so it is unclear if there was any change in his usage of these drugs.

## 3. Discussion

This case report underscores the importance of considering hyperparathyroidism as an organic cause of psychosis, particularly in young patients, even in the presence of substance use disorders. The association between primary hyperparathyroidism and psychiatric symptoms, such as depression, anxiety, cognitive dysfunction, insomnia, confusion, and behavioral changes, was thoroughly discussed during the endocrinology consultation. These manifestations often arise following a prolonged period of subclinical hypercalcemia and vary depending on the severity of hypercalcemia, highlighting the potential impact of elevated calcium levels on mental health [4]. The pathophysiology of these symptoms is thought to be related to glutaminergic excitotoxicity via increased intracellular calcium influx and N-methyl-D-Aspartate (NMDA) receptor activation, resulting in neuronal cell death and subsequent serotoninergic and dopaminergic dysfunction [2].

The patient's history of a similar episode during his college years, which resolved spontaneously without psychiatric intervention, suggests that he may have been experiencing subclinical hypercalcemia for an extended period. This prolonged exposure to elevated calcium levels could have contributed to the severity of his current presentation. Additionally, the patient's concomitant use of cannabis may have further complicated the clinical picture, as substance use disorders can exacerbate or even mimic psychotic symptoms. However, the resolution of psychotic symptoms following parathyroidectomy and normalization of serum calcium levels strongly suggests that PHPT was the primary driver of his psychiatric manifestations.

This case is particularly noteworthy due to the patient's young age at presentation and the severity of his psychotic symptoms, which ultimately led to an involuntary commitment. While psychosis secondary to PHPT has been reported in the literature, it is more commonly observed in older patients, typically in their 50s or 60s [5]. In individuals under the age of 40 years, PHPT is more frequently attributed to uncommon genetic disorders that run in families. These inherited conditions typically result in the enlargement of multiple parathyroid glands, as opposed to the more common scenario of a single affected gland. Certain familial hyperparathyroidism syndromes may also involve dysfunction in other hormone-producing glands throughout the body, such as the pituitary, thyroid, pancreas, or adrenal glands, in addition to the parathyroid glands.

This case emphasizes the difficulty in management of neuropsychiatric changes secondary to prolonged hypercalcemia. Although the patient showed significant clinical improvement with the use of olanzapine, a second-generation antipsychotic, and lorazepam, a benzodiazepine, some symptoms persisted by the end of inpatient treatment. These lingering symptoms may be attributed to irreversible neuronal damage caused by an extended period of hypercalcemia, as suggested by the patient's history of multiple kidney stones.

Although olanzapine was effective in managing the patient's symptoms, evidence suggests that definitive treatment of PHPT through surgery may reduce symptom severity or ameliorate them entirely. Paranoid psychosis and delusions in the setting of hypercalcemia often remit upon normalization of serum calcium or treatment of parathyroid adenoma, and it is recommended that those who experience severe psychiatric symptoms such as psychosis in PHPT undergo surgery for parathyroidectomy [6, 7].

The CT and MRI scans revealed calcifications in the fourth ventricle, an intriguing finding that raises questions about the potential long-term effects of prolonged hypercalcemia on the central nervous system. Although the significance of these calcifications in relation to the patient's psychiatric symptoms remains unclear, future research could explore the association between brain calcifications and neuropsychiatric manifestations in patients with PHPT.

An interesting aspect of this case is the patient's background in physics and his fixation on a specific “mathematical symbol” during his psychotic episodes. While the content of psychotic symptoms varies widely among individuals, the patient's preoccupation with abstract concepts related to his field of expertise is noteworthy. This may reflect the interplay between his preexisting knowledge and the neurochemical disturbances caused by hypercalcemia.

The patient's failure to follow-up with the recommended endocrinology consultation after his initial neurological workup in 2021 raises concerns about the continuity of care and potential barriers to accessing specialized medical services. This case highlights the importance of patient education, effective communication between healthcare providers, and the need for a multidisciplinary approach in managing complex cases involving both psychiatric and endocrine disorders.

When considering the potential weaknesses of this case, several factors should be considered. The resolution of psychotic symptoms following parathyroidectomy and normalization of serum calcium levels strongly suggests a causal relationship between PHPT/hypercalcemia and the patient's psychiatric manifestations. However, the patient's family history of bipolar disorder, long-standing cannabis use, and psychosocial stressors cannot be overlooked as potential contributors to a coincidental psychiatric disorder. To strengthen the case for psychosis secondary to PHPT/hypercalcemia, a more detailed timeline of the patient's psychiatric symptoms in relation to the onset and progression of hypercalcemia and PHPT, a comprehensive assessment of the patient's psychiatric status following parathyroidectomy, and a thorough exploration of the patient's substance use history would provide clearer insights into the relationship between the two conditions. A comprehensive evaluation and long-term follow-up of the patient's psychiatric status would also help to further elucidate the relationship between PHPT/hypercalcemia and psychosis in this case.

## 4. Conclusion

In conclusion, this case shows the complex relationship between hyperparathyroidism, hypercalcemia, and neuropsychiatric manifestations, including psychosis. While the precise pathophysiological mechanisms underlying the psychiatric symptoms associated with PHPT remain hypothetical, recognizing this association has significant implications for diagnosis and treatment. Prompt identification of hyperparathyroidism and appropriate management through endocrine evaluation and intervention are essential for improving patient outcomes. This case underscores the importance of considering organic causes in patients presenting with psychiatric symptoms and highlights the need for a multidisciplinary approach in managing complex cases involving both endocrine and psychiatric disorders.

## Data Availability

The case report data used to support the findings of this study are included within the article.

## References

[1] Radua J., Ramella-Cravaro V., Ioannidis J. P. A. (2018). What causes psychosis? An umbrella review of risk and protective factors. *World Psychiatry*.

[2] Nagy L., Mangini P., Schroen C., Aziz R., Tobia A. (2020). Prolonged hypercalcemia-induced psychosis. *Case Reports in Psychiatry*.

[3] Singh P., Bauernfreund Y., Arya P., Singh E., Shute J. (2018). Primary hyperparathyroidism presenting as acute psychosis secondary to hypercalcaemia requiring curative parathyroidectomy. *Journal of Surgical Case Reports*.

[4] Parks K. A., Parks C. G., Onwuameze O. E., Shrestha S. (2017). Psychiatric complications of primary hyperparathyroidism and mild hypercalcemia. *American Journal of Psychiatry*.

[5] Fuleihan G. E.-H., Chakhtoura M., Cipriani C. (2022). Classical and nonclassical manifestations of primary hyperparathyroidism. *Journal of Bone and Mineral Research*.

[6] Rosenthal M., Gil I., Habot B. (1997). Primary hyperparathyroidism: neuropsychiatric manifestations and case report. *The Israel Journal of Psychiatry and Related Sciences*.

[7] Otsuki K., Izuhara M., Miura S. (2021). Psychosis in a primary hyperparathyroidism patient with mild hypercalcemia: a case report. *Medicine*.

